# Patterns of Presentation of SARS-CoV-2 Infection in Children. Experience at the Italian Epicentre of the Pandemic

**DOI:** 10.3389/fped.2021.629040

**Published:** 2021-01-28

**Authors:** Angelo Mazza, Angelo Di Giorgio, Laura Martelli, Ciretta Pelliccia, Moira Alessandra Pinotti, Vera Quadri, Lucio Verdoni, Alice Decio, Maurizio Ruggeri, Lorenzo D'Antiga

**Affiliations:** ^1^Paediatric Pulmonology, Paediatric Department, Hospital Papa Giovanni XXIII, Bergamo, Italy; ^2^Paediatric Gastroenterology, Paediatric Department, Hospital Papa Giovanni XXIII, Bergamo, Italy; ^3^Paediatric Nephrology, Paediatric Department, Hospital Papa Giovanni XXIII, Bergamo, Italy; ^4^Paediatric Endocrinology, Paediatric Department, Hospital Papa Giovanni XXIII, Bergamo, Italy; ^5^Paediatric Allergology, Paediatric Department, Hospital Papa Giovanni XXIII, Bergamo, Italy; ^6^Paediatric Rheumatology, Paediatric Department, Hospital Papa Giovanni XXIII, Bergamo, Italy; ^7^Child Neuropsychiatry Service, Hospital Papa Giovanni XXIII, Bergamo, Italy; ^8^Paediatric Department, Hospital Papa Giovanni XXIII, Bergamo, Italy

**Keywords:** COVID-19, children, clinical manifestation, SARS–CoV−2, immunity

## Abstract

**Background:** COVID-19, a disease caused by the new coronavirus SARS-CoV-2, spread worldwide, and Bergamo was one of the most affected areas in Europe. Following the first outbreak, more than half of the population of the Bergamo province had been infected. We aimed to describe the patients admitted to our unit shortly after the first outbreak.

**Methods:** we retrospectively reviewed the notes of all pediatric patients diagnosed with COVID-19. We enrolled patients with positive swabs or serology and classified them based on the pattern and the timing of presentation after the first outbreak. This setting was considered a reliable reflection of the consequences of unmitigated SARS-CoV-2 circulation.

**Results:** We diagnosed 35 patients over a 3-month period and we identified six patterns presenting in two temporal phases: Early phase, Group 1 (median of 20 days from epidemic start, IQR: 15–27): neonatal sepsis (*n*.7), pneumonia (*n*.5), flu-like symptoms (*n*.2). Late phase, Group 2 (59:51–66 days, *p* < 0.001): MIS-C (*n*.18), neurological manifestations (*n*.3). Group 1 differed from Group 2 for younger age (1 vs. 8 years, *p* = 0.02), lower C-reactive protein (0.9 vs. 16.6 mg/dl, *p* = 0.008), procalcitonin (0.16 vs. 7.9 ng/ml, *p* = 0.008) and neutrophil count (3,765 vs. 6,780/μl, *p* = 0.006), higher rate of positive swabs (14/14 vs. 9/21, *p* < 0.001), higher lymphocyte count (3,000 vs. 930/μl, *p* = 0.006) and platelet count (323,000 vs. 210,000/μl, *p* = 0.009).

**Conclusions:** Following an outbreak of unmitigated SARS-CoV-2 diffusion, infected children may present with clinical patterns suggesting two temporal clusters, the first characterized by markers of direct viral injury, the second suggesting an immune-mediated disease.

## Introduction

The SARS-CoV-2 epidemic, causing a disease named “COVID-19,” has spread worldwide since the beginning of 2020. Italy was the first European country to be involved and Bergamo, the most affected city in the first wave of the epidemic. In Italy 960,373 infected people and 41,750 deaths have been reported as of November 9. Overall 265,531 cases occurred in Lombardy, of which 20,390 in Bergamo (1). Shortly after the free circulation of the virus, a local study revealed that 56% of the population of the Bergamo province had been infected by SARS-CoV-2 (2).

These numbers argue why Bergamo and its area (around 1,200,000 inhabitants in all) have become a model of the impact of COVID-19 epidemic on people's health.

COVID-19 presents different patterns of clinical presentation in adults and children (3). In adults, the clinical picture is dominated by interstitial pneumonia possibly complicated by cardiovascular involvement. Following the infection of an adult person, a three-phase course of the disease has been described, with a progression from initial involvement of the upper airways to abnormal chest imaging due to the descent of the virus to the alveolar cells; the third phase is characterized by a vigorous immune response of the host, leading to a cytokine storm, with elevated inflammatory and cardiac biomarkers associated with severe interstitial pneumonia (4).

In children the clinical presentation is mild, mostly with an influenza-like pattern (5). Serious cases are rare and full recovery is the rule. Few cases require hospitalization, but the majority of children can be managed as outpatients or have an asymptomatic course (5, 6).

The aim of this study is to analyze the clinical pattern of pediatric patients admitted to our unit during the first 3 months of the outbreak of SARS-CoV-2 in Bergamo, following a period of unmitigated viral circulation.

## Patients and Methods

### Study Setting

In this study we retrospectively reviewed the notes of patients admitted to the general paediatric unit of Hospital Papa Giovanni XXIII in Bergamo (Italy), and managed as inpatients, from February 25 to May 23, 2020. Our unit is a tertiary pediatric referral center with ~1,300 pediatric admissions per year, serving a province of approximately one million people.

From the start of the epidemic we set up a dedicated COVID area in our general pediatric ward, hosting all patients/parents with a positive nasopharyngeal/oropharingeal (NP/OP) swab, managed with full isolation and personal protective equipment (PPE) (7).

No other hospitals in our area created a COVID-19 dedicated area. The patients were admitted in the first phase of the epidemic and during the lockdown, therefore we considered this situation a reliable reflection of the consequences of unmitigated SARS-CoV-2 circulation in the pediatric population of the province of Bergamo.

### Confirmation of SARS-CoV-2 Infection

During the study period all patients admitted to our unit underwent NP/OP, testing SARS-CoV-2 nucleic acid using real-time reverse-transcriptase polymerase-chain-reaction (RT-PCR) assay; patients with a positive NP/OP test were considered confirmed cases of SARS-CoV-2 infection.

A proportion of patients admitted to our unit underwent also a test for the qualitative detection of SARS-CoV-2 antibodies (IgM and IgG) through a lateral flow chromatographic immunoassay (NADAL® COVID-19 IgG/IgM Test, nal von minden GmbH, Carl Zeiss Strasse 12, 47445 Moers, Germany). Positivity of IgM and/or IgG was considered consistent with a recent or previous infection by SARS-CoV-2, respectively.

### Definitions

Among all admissions we identified those who were related to SARS-CoV-2 infection based on NP/OP and serology testing (here referred to as “COVID-19”). COVID-19 patients were analyzed to ascertain different patterns of presentation by age and symptoms. We classified COVID-19 patients into six categories, based on previous reports on manifestations of SARS-CoV-2 in children (8).

- *Neonatal/Infantile viral sepsis:* patients meeting the criteria for paediatric systemic inflammatory response syndrome (SIRS) (at least two from fever, tachycardia, tachypnea, elevated leukocyte count; one of two must be fever or abnormal leukocyte count) in children under 3 months of age (9).
- *Pneumonia:* presence of a compatible chest X ray.
- *Influenza-like syndrome:* history of fever and upper respiratory tract symptoms.
- *Multisystem inflammatory syndrome in children (MIS-C):* Age <21 years and a presentation including all of the following:
  ° Fever >38.0°C for ≥24 h, or report of subjective fever lasting ≥24 h;
  ° Laboratory evidence of inflammation including one or more from: elevated C-reactive protein, erythrocyte sedimentation rate, fibrinogen, procalcitonin, D-dimer, ferritin, lactic acid dehydrogenase, or interleukin-6 [IL-6] level, elevated neutrophils, reduced lymphocytes and low albumin;
  ° Severe illness requiring hospitalization;
  ° ≥2 organ systems involved (cardiac, renal, respiratory, hematologic, gastrointestinal, dermatologic, and/or neurologic);
  ° No alternative plausible diagnoses;
  ° Recent or current SARS-CoV-2 infection or exposure, defined as **any** of the following: Positive SARS-CoV-2 polymerase chain reaction (PCR); Positive serology for SARS-CoV-2; Positive antigen test; COVID-19 exposure within the 4 weeks prior to the onset of symptoms (10).
- *Neurological manifestations:* any symptoms related to nervous system involvement requiring hospitalization.
- *Miscellaneous*: NP/OP positive for SARS-CoV-2 but admitted for reasons unrelated to COVID-19 (surgical problems, trauma, chemotherapy or skin rash) and managed in our pediatric COVID area.

### Clinical, Laboratory and Radiological Evaluation

Data obtained from the hospital medical records populated an *ad hoc* database and included demographic informations, presenting symptoms, contact with confirmed or suspected cases of COVID-19, laboratory data (including white blood cell count, neutrophils and lymphocyte count, C-reactive protein (CRP), pro-calcitonin, lactate dehydrogenase (LDH), Alanine aminotransferase (ALT) and creatinine). Chest X-ray was evaluated when performed.

### Statistical Analysis

The Student *t*-test, the χ2 method, or Fisher's exact test were performed when appropriate to compare continuous and categorical variables. A *P*-value < 0.05 was chosen as cut-off for significance. Data were analyzed with SPSS (IBM Corp. Released 2011. IBM SPSS Statistics for Mac, Version 20.0. Armonk, NY: IBM Corp) and GraphPad Prism (GraphPad Prism version 5.00 for Mac, GraphPad Software, San Diego, CA) softwares. The study received the approval of the Bergamo Ethics Committee within the frame of the proposal entitled “Retrospective observation for a better understanding of epidemiology and outcome of COVID-19 disease”(registration n. 37/20, 25/03/2020).

## Results

### Study Group

**Figure 1:**
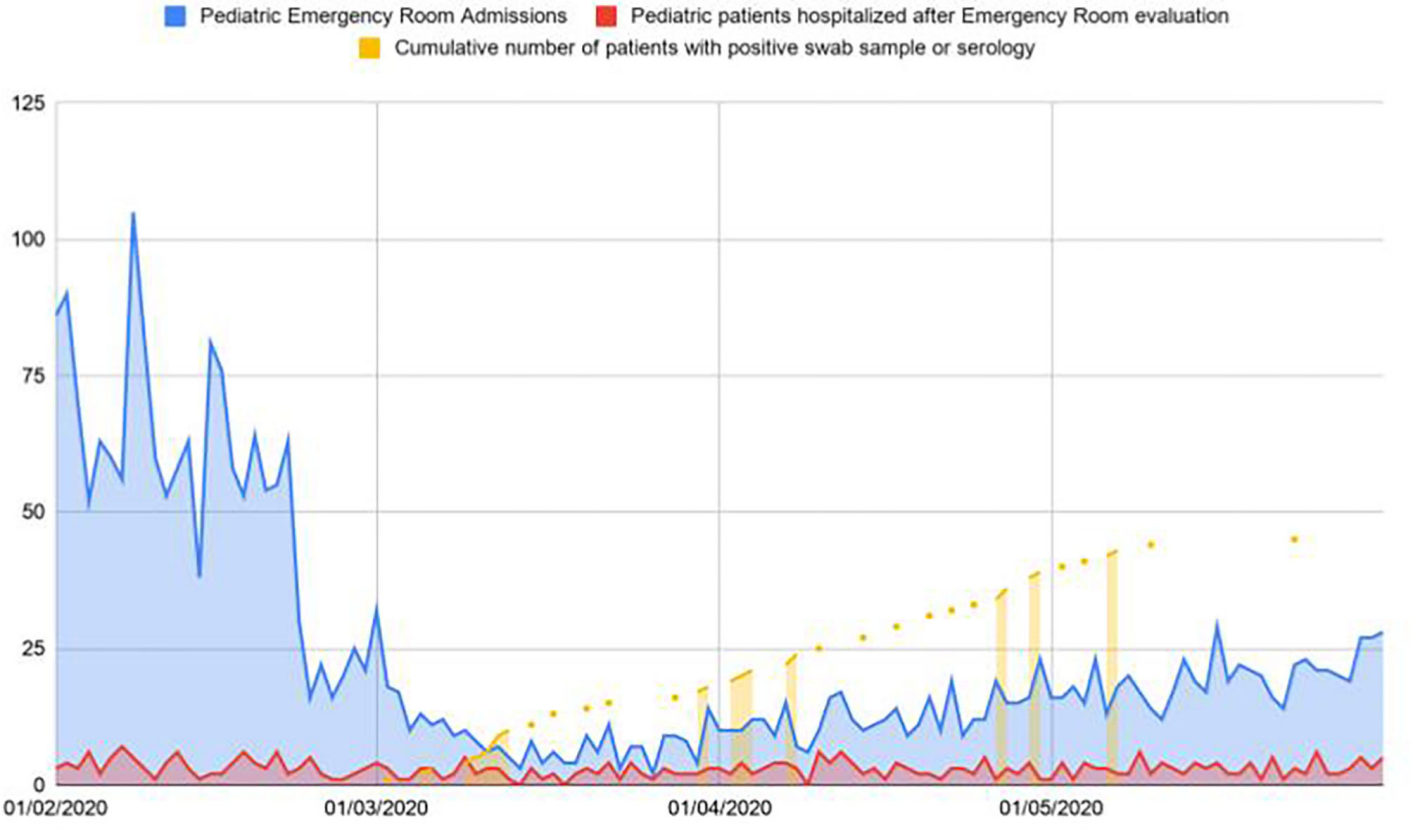
Patients visiting our emergency department in the first 3 months of the pandemic in comparison with patients hospitalized and testing positive for SARS-CoV-2.

From February 25 to May 23, 153 patients were admitted to our unit. In the same period of the previous year we admitted 173 patients. All the 153 patients performed a NP/OP for SARS-CoV-2 and 33 resulted positive. Twenty patients of the 153 also performed a serological test with a positive result in 15. Three of these patients had a positive NP/OP sample as well. Figure 1 reports data regarding patients visiting our emergency department, providing information on percentage of hospitalizations and discharge.

Overall, 45/153 (32% of all admitted) patients were diagnosed with COVID-19 presenting as: viral sepsis (*n*. 7), pneumonia (*n*. 5), flu-like symptoms (*n*. 2), MIS-C (*n*. 18), neurological manifestations (*n*. 3). The remaining 10 patients were admitted to our pediatric COVID-19 area because of the positive result of the NP/OP swab screening, but without a clinical pattern in keeping with a viral infection or SARS-CoV-2 immune-mediated disease. Six out of 45 patients were admitted to Intensive Care Unit (ICU), none of them died.

**Figure 2:**
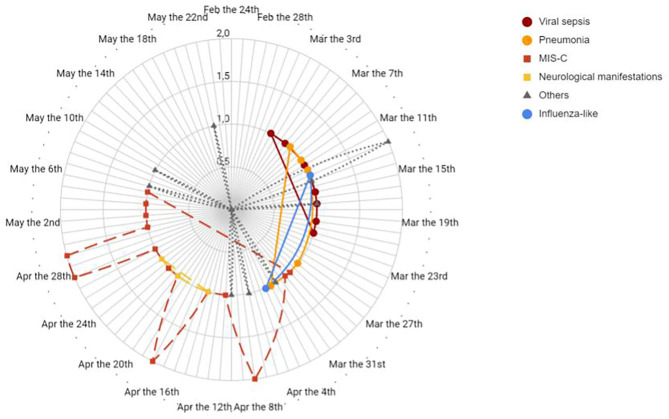
Timeline distribution of patients divided by clinical pattern of presentation diagnosis. Solid line indicates diagnosis of viral sepsis, pneumonia and influenza like symptoms in different shades of grey. Dashed line indicates diagnosis of MIS-C and neurological manifestations. Dotted line indicates other diagnosis.

Figure 2 reports the timeline distribution of the various patterns of presentation of our patients.

**Figure 3:**
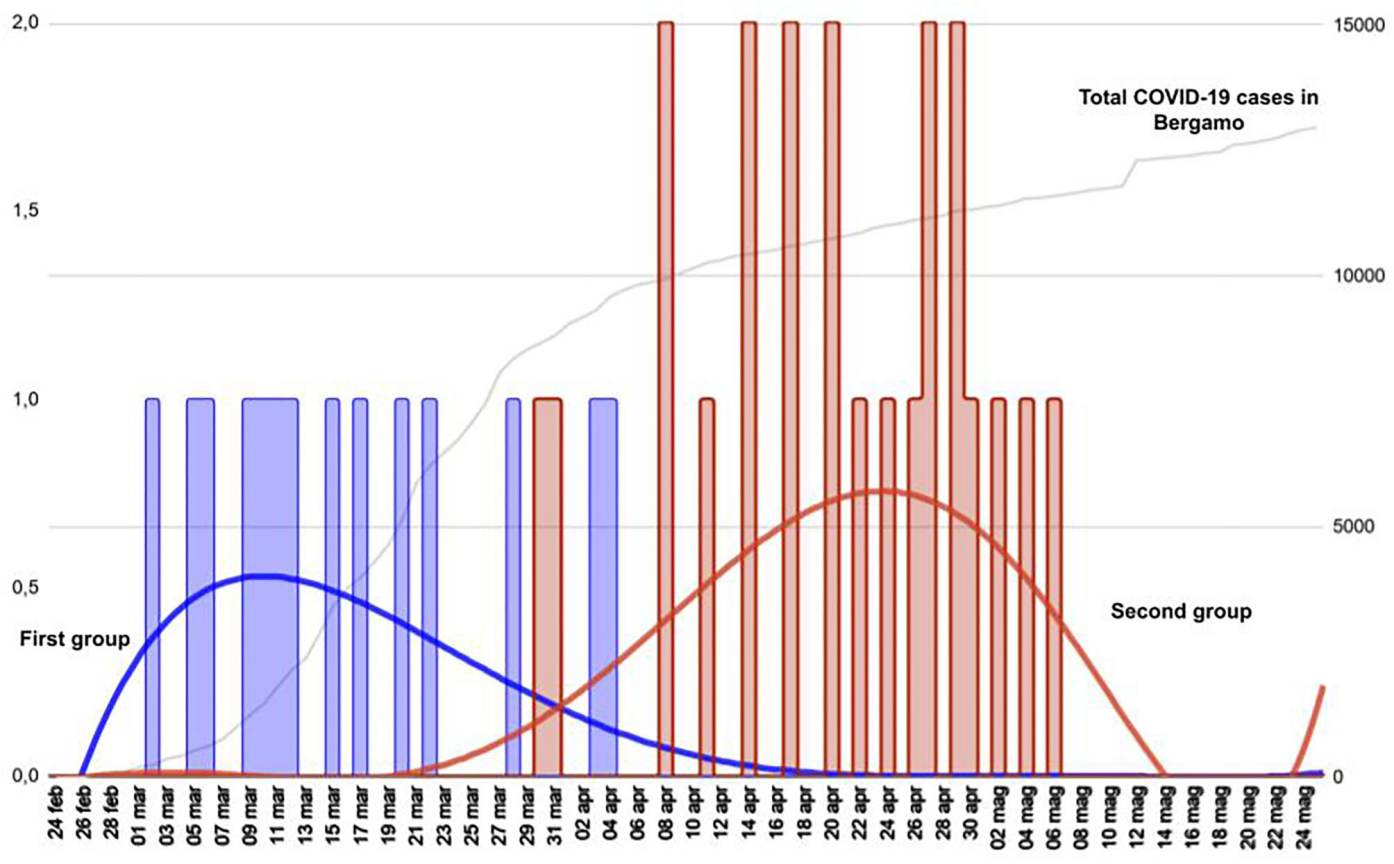
Timeline distribution of cases of COVID-19 showing a clearly biphasic pattern of presentation. Total COVID-19 cases in Bergamo depicts the epidemic trend in the city. The first group comprises children with viral sepsis, pneumonia and influenza-like sdr, whereas the second group comprises patients with MIS-C and neurological manifestations.

**Figure 4:**
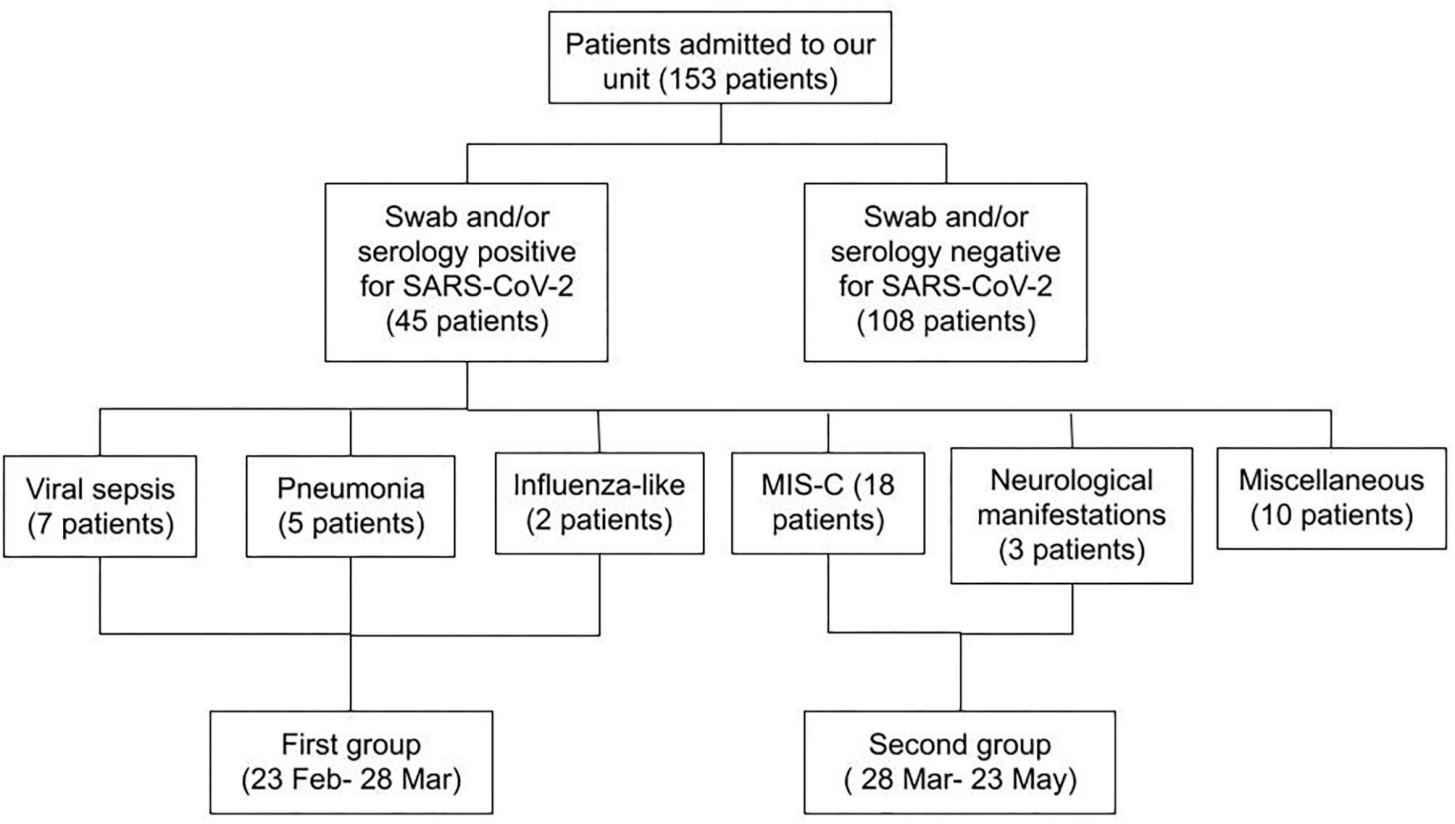
Flow chart showing the features of all patients admitted during the study period. A first differentiation was made based on swab and serology results for SARS-CoV-2. Patients with a positive result of one or both tests were divided according to the pattern and the timing of presentation.

Figure 3 shows that the different patterns of presentation can be grouped based on the different timing of onset. All patients with viral sepsis, pneumonia and influenza-like symptoms (14 children) presented in the first month following the start of the local epidemic, dating February 23 (median time from the epidemic onset 20 days, IQR 15–27), and are classified as Group 1. Patients with a diagnosis of MIS-C or neurological manifestations (21 children) presented in the second and third month after the start of the epidemic (median time 59 days, IQR 51–66) and are classified as Group 2 (Group 1 vs. Group 2 *p* < 0.001). The 10 patients with an incidentally positive NP/OP, admitted for conditions unrelated to COVID-19, were distributed evenly in the study period and are not included in this analysis (Figure 4). Data on all the patients are reported also in Table 1.

**Table 1:** Demographic and clinical features of all patients observed in the study period.

	**Pt**.	**Age (ys)**	**Weight (Kg)**	**Sex**	**Ethnicity**	**NP/OP**	**Serology**	**Familiar contact**	**Diagnosis**	**Days of fever**	**WBC (10^**∧**^9/L)**	**NEU (10^**∧**^9/L)**	**Lymph (10^**∧**^9/L)**	**HB (g/L)**	**PLT 10^**∧**^9/L**	**CRP (mg/dl)**	**PCT (ng/ml)**	**LDH (IU/L)**	**Creat (mg/dl)**	**ALT (IU/L)**	**CXR**	**Treatment**	**Notes**
Group 1	1	0.1	3.4	M	C	+	N/A	-	Sepsis	3	7390	4030	1470	15.5	200	0.7	0.24	850	0.31	99	patchy infiltrates	ATB	
	2	0.1	3.6	F	C	+	N/A	-	Sepsis	2	7910	2470	4170	13.8	278	0.5	0.05	440	0.26	20	Perihilar opacities	ATB	
	3	0.1	4	M	C	+	N/A	+	Sepsis	3	7230	3100	3600	8.9	726	<0,05	<0.05	310	0.31	27	N/A	ATB	
	4	0.1	3.6	M	C	+	N/A	-	Sepsis	0	11080	2670	5150	13.7	322	0.1	0.09	367	0.27	34	Negative	ATB + HFO	ICU admittance
	5	0.2	5.8	F	C	+	N/A	-	Sepsis	4	7400	2400	3600	13.1	344	0.1	0.35	365	0.22	53	N/A	ATB	
	6	0.2	3.8	M	C	+	N/A	+	Sepsis	1	8050	2690	5200	10.3	340	0.2	0.15	318	0.21	33	N/A	ATB	
	7	0.1	4.9	F	SA	+	N/A	+	Sepsis	5	8620	4500	3000	11.5	622	0.3	3.45	402	0.2	19	N/A	ATB	
	8	14.7	80	F	C	+	+	+	pneumonia	15	7440	6570	500	13.3	166	15.1	0.6	721	1.35	41	Perihilar opacities	CPAP, IOT, ATB, heparin	ICU admittance
	9	6.6	19	F	C	+	N/A	+	pneumonia	5	5390	3500	1200	4.6	189	6.8		216	1.36	9	Perihilar opacities	ATB, NIV	
	10	9.7	35	F	As	+	N/A	+	pneumonia	14	6740	4800	1080	9.5	179	11	0.71	415	0.7	14	Lobar pneumonia, pleural effusion	ATB. Steroid	Mycoplasma +
	11	4	14	M	C	+	N/A	+	pneumonia	0	14140	9690	3490	13.3	351	0.05		353	0.38	20	Lobar pneumonia	ATB. Steroid	Parainfluenzae +
	12	14.7	37	M	C	+	N/A	+	pneumonia	6	8250	4240	2690	13.2	449	0.9	1.6	438	0.42	47	Perihilar opacities	NIV, antiviral, hydroxicloroquine, ATB	CKD
	13	7.2	21	M	C	+	N/A	+	influenza-like	3	5800	3000	1700	13.5	250	0.1	0.13	631	0.44	24	Negative	NSAID	Metapneumovirus +, ICU admittance
	14	1.1	11.8	M	C	+	N/A	+	influenza-like	2	9360	5850	3000	10.7	324	0.4	0.42	387	0.29	24	Negative	NSAID	
Group 2	15	7	45	M	C	+	+	+	MIS-C	7	12730	11260	1000	13.4	130	30	59	729	0.8	79	Perihilar opacities	ATB, IVIG, ASA, steroid	Meningeal signs
	16	2.9	13.9	F	C	-	+	-	MIS-C	6	4480	3250	860	11	116	12.2	6.6	424	0.28	46	Perihilar opacities	ATB, IVIG, ASA, steroid	
	17	7.6	31.8	F	C	+	+	+	MIS-C	8	10260	9150	670	13.3	228	52.5	7.57	1057	0.5	78	Negative	ATB, IVIG, ASA, steroid	Cardiogenic shock, ICU admittance
	18	5	23	M	C	-	+	+	MIS-C	8	6470	4750	1200	12.1	206	12.7		301	0.42	21	Negative	ATB, IVIG, ASA, steroid	Meningeal signs
	19	5.5	16.5	M	C	-	+	+	MIS-C	8	7280	6060	740	12.6	179	15		318	0.44	20	Negative	ATB, IVIG, ASA, steroid	
	20	9.3	29	M	SA	-	+	-	MIS-C	10	16710	14810	860	11.1	210	24.2	27	302	1.02	63	Lobar pneumonia	ATB, IVIG, ASA, steroid, diuretic	Cardiogenic shock
	21	2.6	12	F	C	+	N/A	+	MIS-C	5	12220	5660	3700	10.6	250	15	5.04	314	0.23	18	Negative	ASA	
	22	5.4	16.5	M	C	-	+	+	MIS-C	7	4660	3860	460	9.3	142	12.2	10.4	219	0.3	20	Lobar pneumonia	ATB, IVIG, ASA, steroid, heparin	Cardiogenic shock
	23	6.9	25	F	Afr	+	N/A	-	MIS-C	6	23120	19559	1600	11.4	237	37.3	64	642	0.27	22	Perihilar opacities	ATB, IVIG, ASA, steroid, heparin	Cardiogenic shock, ICU admittance
	24	1.2	10.6	F	C	-	+	-	MIS-C	7	20140	11000	4480	9.9	795	20		550	0.18	29	Perihilar opacities	ATB, IVIG, ASA, steroid	
	25	17.2	70	M	SA	-	+	-	MIS-C	5	7980	6780	760	15.2	237	15.6		240	1.28	21	Negative	ASA, ATB	
	26	9.2	29	M	C	+	N/A	+	MIS-C	6	12140	9000	520	13.3	142	26.7	8.2	269	0.5	56	Lobar pneumonia	ATB, IVIG, ASA, steroid, diuretic	
	27	6	18	M	C	-	+	+	MIS-C	5	8810	6700	870	11.1	132	26.2	18.1	444	0.31	63	Negative	ATB, IVIG, ASA, steroid, diuretic, inotropes	Cardiogenic shock, ICU admittance
	28	7.6	22	M	C	-	+	+	MIS-C	8	19340	15700	2230	10.1	389	17.6	2.64	221	0.46	52	Negative	IVIG, ASA, ATB	Cardiogenic shock
	29	11.6	69	M	C	-	+	+	MIS-C	5	9900	7800	610	12.1	203	30.5	2.11	302	0.4	24	Perihilar opacities	ATB, IVIG, ASA, steroid, diuretic	Cardiogenic shock
	30	1.8	10.4	F	C	-	+	-	MIS-C	5	13720	9500	2730	9.6	358	9.6	2.87	248	0.28	19	Lobar pneumonia	IVIG, ASA, ATB	
	31	8.3	27	F	C	+	N/A	+	MIS-C	4	5540	4400	430	10	78	13.5	32.8	342	0.46	34	Perihilar opacities and pleural effusion	ATB, IVIG, ASA, steroid	
	32	16.4	52	F	C	+	N/A	-	MIS-C	3	9610	7540	930	12.3	270	18		137	0.59	13	Negative		
	33	17.8	70	M	C	-	+	+	encephalitis	2	4080	1800	1730	15.4	201	0		305	1.28	16	N/A	ATB, antiviral agent, NSAID	
	34	16.3	58	M	C	+	N/A	-	epilepsy	0	8510	5770	2340	15	320	0.05		203	1.09	27	N/A		
	35	12.5	79	F	C	+	N/A	-	IIH	0	7930	3760	3400	12.5	289	0.3		215	0.55	23	N/A	diuretic	
Others	36	0.4	5.4	F	C	+	N/A	-	Solid tumor	3	330	110	210	11.1	75	10.3		453	0.20	21	Lobar pneumonia	ATB	
	37	6.5	17.1	M	C	+	N/A	-	Solid tumor	0	11220	10330	730	9.4	316	0.3		260	0.26	48	N/A	chemotherapy	
	38	14.5	58.5	F	C	+	N/A	-	Solid tumor	7	630	220	400	9.5	25	1.5		167	1.13	31	Perihilar opacities	chemotherapy, ATB, G-CSF	
	39	11.2	43	M	C	+	N/A	+	Appendicitis	1	9860	8110	980	13.9	184	0.6		251	0.54	27	N/A	ATB	
	40	15.4	60	M	C	+	N/A	-	Appendicitis	2	13230	10790	1800	14.5	230	0.2		196	0.57	32	N/A	ATB	
	41	16.4	56	M	C	+	N/A	+	Vasculitis	0	4230	1670	1900	14.5	162	0		146	1.25	17	N/A	NSAID	
	42	3.2	17.3	F	C	+	N/A	-	Leukemia	3	370	30	320	8.4	42	0		381	0.22	181	N/A	ATB	
	43	3.3	15	M	Afr	+	N/A	+	Eye trauma	0	7530	4740	1850	9.3	591	<0.05		478	0.28	23	N/A		
	44	6.9	24	M	Afr	+	N/A	-	Solid tumor	0	7790	3940	3030	11.2	282	0		373	0.37	47	N/A	surgery	
	45	7.9	23	F	C	+	N/A	+	Leukemia	5	40010	200	21800	8.5	8	7.7	1.16	628	0.48	20	Perihilar opacities	chemotherapy, steroid	

*Pt, patient; ys, years; Kg, kilograms; NP/OP, nasopharyngeal/oropharingeal swab; WBC, white blood cells (n.v. 6,000–17,000 10^∧^9/L); Neu, neutrophils (n.v. 1,500–9,000 10^∧^9/L); Lymph, lymphocytes (n.v. 3,000–10,000 10^∧^9/L); HB, hemoglobin (n.v. 12–14 g/L); PLT, platelets (n.v. 150–400 10^∧^9/L); CRP, C-reactive protein (n.v. 0–1.0 mg/dl); PCT, procalcitonine (n.v. < 0.05 ng/ml); LDH, lactate dehydrogenase (n.v. 120–246 IU/L); Creatm, creatinine (n.v. 0.1–0.7 mg/dl); ALT, alanine aminotransferase (n.v. 7–40 IU/L); CXR, chest X-ray; M, male; F, female; C, Caucasian; SA, South American; As, Asian; Afr, African; N/A, not available; MIS-C, multisystem inflammatory syndrome in children; IIH, idiopathic intracranial hypertension; ATB, antibiotic; HFO, high flow oxygen; ICU, intensive care unit; CPAP, continuous positive airway pressure; IOT, orotracheal intubation; NIV, non-invasive ventilation; NSAID, non-steroidal anti-inflammatory drug; IVIG, intravenous immunoglobulin; ASA, acetylsalicylic acid; CKD, chronic kidney disease; G-CSF, granulocyte colony stimulation factor*.

### Group 1: Patients Presenting in the Early Phase

The first cluster presented at a median time from the start of the epidemic of 20 days (Interquartile range, IQR: 15–27) and comprised 14 children with viral sepsis (n. 7), pneumonia (n. 5), and influenza-like sdr (n. 2), of a median age of 1 year (0,1–7) and a median weight of 21 kg (16.5–36). Eight were males (57%) and 12 of Caucasian origin (86%). All patients resulted NP/OP positive for SARS-coV-2. One of them performed a serology test that resulted positive 2 months after diagnosis. A positive history of contact with an affected family member was present in 10 of them (71%). The median days of fever were three (2–5). Medians of the blood count values were, respectively: White blood cells (WBC) 7,675/μl (7,270–8,527), Neutrophils 3,765/μl (2,767–4,725), Lymphocytes 3,000/μl (1,527–3,600), Haemoglobin (Hb) 13.1 g/L (9-5-13.3), platelets 323,000/μl (212,000–349,000). Median CRP was 0.9 mg/dl (0.25–8.9) and median procalcitonin was 0.16 ng/ml (0.11–0.51). Median LDH was 395 IU/L (356–440), creatinine 0.34 mg/dl (0.2–0.4) and ALT 26 IU/L (20–39). A chest X-ray was obtained in 10 patients and resulted positive in five with pneumonia and two with neonatal sepsis. Eight of 10 were treated with a course of intravenous antibiotics, three needed invasive respiratory support and one received antiviral therapy. Anti-inflammatory medications (steroid and hydroxychloroquine) were administered to 3 out of 14 patients.

### Group 2: Patients Presenting in the Late Phase

The second group presented at a median time from the start of the epidemic of 59 days (51–66), and comprised 21 children with MIS-C (n. 18) and neurological manifestations (n. 3), of a median age of 8 years (5.4–11.5) and a median weight of 28 kg (17.6–53.5). Twelve were males (57%), 18 of Caucasian origin (86%). Nine patients had a SARS-CoV-2 positive NP/OP (43%) and 12/21 (57%) had a positive serology; two patients were positive for both. A positive history of contact with an affected family member was present in 12 of them (57%). The duration of fever was a median of 6 days (5–7). The median of the blood count values were, respectively: WBC 9,610/μl (7,280–12,730), Neutrophils 6,780/μl (4,750–9,500), Lymphocytes 930/μl (740–2,230), Hb 12.1 g/L (10.5–13.3), Platelets 210,000/μl (142,000–270,000). Other blood tests showed: CRP 16.6 mg/dl (12.9–25.7), procalcitonin 7.9 ng/ml (4.5–28.5), LDH 302 IU/L (240–424), creatinine 0.45 mg/dl (0.2–0.4) and ALT 24 IU/L (20–52). Chest X-ray was performed in 18 patients and tested positive in nine cases. Fifteen patients were treated with intravenous immunoglobulin (IVIG) and acetyl salicylate acid (ASA), 13 with steroids and 16 with an antibiotic course.

### Miscellaneous

This group presented at a median time from the start of the epidemic of 42 days (20–67), and comprised 10 patients with a median age of 7 years (4–14) and a median weight of 24 Kg (17–53). They were admitted for surgical, haematological or dermatological illnesses. Six were males (60%) and 8 were of Caucasian origin (80%). All these patients had a positive NP/OP for SARS-CoV-2 and four of them had a history of contact with a intrafamiliar positive case. Median days of fever was two (0–3). Median of the blood exams was: WBC 7,660/μl (1,530–10,880), Neutrophils 2,805/μl (205–7,268), Lymphocytes 1,390/μl (483–1,888), haemoglobin 10.3 g/L (9.4–11.2), platelets 173,000/μl (50,000–269,000), CRP 0.15 mg/dl (0–1.2), LDH 317 IU/L (210–435), creatinine 0.42 mg/dl (0.02–0.04) and ALT 29 IU/L (22–43) respectively. Three patients performed a chest X-ray, consistent with pneumonia in two of them. The third patient had a negative chest X-ray but performed a CT scan resulting positive for interstitial pneumonia.

### Comparison From Group 1 and Group 2

A statistical analysis was carried out to compare Group 1 and Group 2, and data are summarized in Table 2.

**Table 2:** Comparison between the two groups of patients diagnosed with SARS-CoV-2 infection cases.

	**Group 1 (*n* 14)**	**Group 2 (*n* 21)**	***P***
**Presentation after the start of the pandemic (days)**	**20 (15–27)**	**59 (51-66)**	** <0.001**
**Age (years)**	**1 (0.1–7)**	**8 (5.4–11.5)**	**0.02**
Sex (M/F)	8/6	12/9	1
Weight (Kilograms)	21(16.5–36)	28 (17.6–53.5)	0.57
Affected family member	10/14 (71%)	12/21 (57%)	0.48
**Positive swab sample**	**14/14 (100%)**	**9/21 (43%)**	** <0.001**
Positive serology	1/1	14/21 (67%)	-
Fever duration (days)	3 (2–5)	6 (5–7)	0.42
White cell count (n.v. 6,000–17,000/μl)	7,675 (7,270–8,527)	9,610 (7,280–12,730)	0.1
**Neutrophil count (n.v. 1,500–9,000/μl)**	**3,765 (2,767–4,725)**	**6,780 (4,750–9,500)**	**0.006**
**Lymphocyte count (n.v. 3,000–10,000/μl) 10**^**∧**^**9/L)**	**3,000 (1,527–3,600)**	**930 (740–2,230)**	**0.006**
Haemoglobin (n.v. 12–14 g/L)	13.1 (9.5–13.3)	12.1 (10.5–13.3)	0.3
**Platelet count (n.v. 150–400/ml)**	**323 (212–349)**	**210 (142–270)**	**0.009**
**CRP (n.v. 0–1.0 mg/dl)**	**0.9 (0.25–8.9)**	**16.6 (12.9–25.7)**	**0.008**
**Procalcitonin (n.v**. **<** **0.05 ng/ml)**	**0.16 (0.11–0.51)**	**7.9 (4.5–28.5)**	**0.008**
LDH (n.v. 120–246 IU/L)	395 (356–440)	302 (240–424)	0.30
ALT (n.v. 7–40 IU/L)	26 (20–39)	24 (20–52)	0.76
Creatinine (n.v. 0.1–0.7 mg/dl)	0.34 (0.2–0.4)	0.45 (0.2–0.4)	0.34
Chest X ray performed	71%	86%	-
ICU admittance	3/14 (21%)	3/21 (14%)	0.66

The two clusters show a significant difference for time of presentation after the start of the pandemic, age, positive swab sample, Neutrophil, Lymphocyte and platelet count, CRP and procalcitonin. M, male; F, female; WBC, white blood cells; CRP, C-reactive protein; LDH, lactate dehydrogenase; ALT, alanine aminotransferase; ICU, intensive care unit; Data are reported as medians and interquartile range (IQR).

*Bold values evidence significative statistical differences between the two groups*.

Days of presentation after the start of the pandemic differed significantly from the two groups and was delayed in Group 2 (*p* < 0.001), as well as age that was older in Group 2 (*p* = 0.02). There was a significant difference for Neutrophil and platelet count, CRP and procalcitonin, that were all higher in Group 1. A significant difference was found also for Lymphocyte count, lower in Group 2. No significant differences were observed for renal and liver function, LDH levels and number of days of fever.

All patients in Group 1 had a positive swab sample for SARS-CoV-2 against 43% in Group 2 (*P* = < 0.001). Serology was performed in one of Group 1 and was positive (other patients could not perform serology because it was not yet available at the moment), vs. 14 positive on 21 tested in Group 2. A history of contact with a COVID-19 case was present in 10/14 (71%) of Group 1 vs. 12/21 (57%) of Group 2 (*P* = 0.48).

## Discussion

The analysis of the first European centre hit by SARS-CoV-2 pandemic may be advantageous in terms of understanding COVID-19 patterns of presentation in children. Indeed, the fact that at that time the local situation was not mitigated by containment measures and that a lockdown was adopted shortly thereafter makes this setting a reliable frame depicting the effects of SARS-CoV-2 free viral circulation. In this context, the timeline distribution (Figures 2, 3) of the diagnoses related to COVID-19 revealed a biphasic pattern, well-distinguishable in terms of clinical presentation.

In the first phase of the epidemic, infants were admitted with patterns typical of an infectious disease: flu-like symptoms, pneumonia or viral sepsis. All these cases, called Group 1, are attributable to a mechanism of direct injury by SARS-CoV-2, with half of them presenting chest X ray lesions, and all of them having a positive NP/OP for SARS-CoV-2. Intriguingly this “infectious pattern” of presentation disappeared with social isolation measures. No case, with the same characteristics, has occurred since April 4 (in Italy the lockdown started on March 9).

The second group of patients, here referred to as Group 2, comprised 21 children: 18 with a diagnosis of MIS-C and three with neurological manifestations. Only 43% of patients presented a positive NP/OP for SARS-CoV-2, whereas the others had a positive serology. The statistical comparison between the two groups (Table 2) reveals a significant difference in blood cells count (neutrophils, lymphocytes and platelets) and in markers of inflammation (procalcitonin and CRP), suggesting a different pathophysiology of the disease. Remarkably, the first patient with a diagnosis of MIS-C was admitted on the 30^th^ of March while the first adult case of COVID-19 reported in the same region dates back to February 22.

SARS-CoV-2 infection in children usually presents a benign course, when compared to the adult population. All reports published so far show that children mostly present mild respiratory symptoms, with sporadic complications limited to people with comorbidities (11).

Patients admitted to our unit confirmed this impression. The patients with a more severe respiratory involvement had underlying comorbidities (Down syndrome and children with special health care needs). As already reported, several patients had some chest radiological abnormalities without showing any respiratory symptom (12). This data supports the tropism of SARS-CoV-2 for the respiratory tract also in asymptomatic patients (3, 13).

The different expression of angiotensin converting enzyme-2 (ACE-2) receptor in children and adults has been advocated as one of the factors implicated in the reduced morbidity of SARS-CoV-2 observed at young ages (14, 15). ACE-2 is a counter-regulatory enzyme of the renin-angiotensin-system acting by converting angiotensin-2 to Ang-(1–7) form. Upregulation of the angiotensin-2 axis leads to pro-inflammatory effects in the respiratory and cardiovascular systems (16). After entering pneumocytes, SARS-CoV-2 downregulates ACE-2 expression, decreasing angiotensin-2 metabolism. In children a reduced expression of ACE-2 receptor in upper airways may be protective against COVID-19, leading to less severe disease (17).

Sepsis-like presentation in neonates, already reported in literature (18, 19), can be explained by the characteristics of immunity in the first phases of life. Infants present an immature B lymphocyte and Th1 and Th2 response, but harbor a high level of Tregs. The innate immune response represents the first line of protection against SARS-CoV-2, but in case of dysregulation of such response, more typical of older age, this can result in an excessive inflammation leading to a severe disease and even death (20).

Patients with COVID-19 tend to present lymphopenia and high biomarkers of inflammation (21). These findings are prominent in Group 2 of our study, differing significantly from Group 1. Intriguingly these groups differ also by age. The age-related evolution of the immune system can play a role in this difference (22).

The pathophysiology of severe COVID-19 in adults follows different phases of injury with a first phase of infection, usually characterized by direct lung damage, followed by an inflammatory reaction known as “cytokine storm,” causing a rapid clinical deterioration (23, 24). Such condition seems to be different in children. After the onset of the epidemic worldwide, there have been several reports of increased incidence of Kawasaki-like disease cases (25, 26). The Center for Disease Control and Prevention and the World Health Organization proposed a new diagnostic framework, called multisystemic inflammatory syndrome associated to COVID-19 in children (10, 27). The first published case series comes from our experience in the Bergamo province (26).

After the first report of 10 cases, we analyzed 18 patients meeting the criteria for MIS-C. In this setting SARS-CoV-2 may act as a superantigen cascading macrophages and complement recruitment, leading to a sort of “cytokine storm” (23, 28, 29). In adults, the inflammatory cascade seems to be activated in 2–3 weeks (30, 31). All our patients with MIS-C presented in the second and third month (Figures 2, 3) of the epidemic, suggesting that activation latency in children may be longer.

Neurological symptoms associated with SARS-CoV-2 exposure have been reported in adults (32) and in children (33, 34). Some of these symptoms appear to be the consequence of direct viral invasion of the nervous system tissue, others arise as a post viral autoimmune process, or are the result of metabolic and systemic complications due to the associated critical illness (32). Our analysis found three patients with mixed neurological manifestations and two patients with MIS-C and meningeal signs, in the second phase of the epidemic, suggesting a post viral autoimmune process as the cause of the clinical picture.

In the described cohort almost all pediatric ages are represented but clusters are detectable in infants, toddlers and in school age. All cases presenting in infants belong to the pattern of viral sepsis, while patients with respiratory symptoms tend to be toddlers or in school age. MIS-C and neurological patients, presenting in school age, suggest a relationship between immune system maturity and autoimmune complications.

Multicenter studies confirm COVID-19 infection is differently expressed in different pediatric age groups. Infants under 1 year of age represent 29% of infected pediatric patients (7% under 1 month of age), 10% between 1 and 2 years, 11% between 2 and 5 years, 16% between 5 and 10 years and 34% over 10 years (35). The hospitalization rate in the pediatric population is around eight per 100,000 (36). Our Covid-19 pediatric area was the only one in a region of about 300,000 pediatric subjects, suggesting a hospitalization rate of 15 out of 100,000. This data suggests the relevance of our analysis in a period of free viral circulation with more than 50% of the population demonstrating antibodies for SARS-CoV-2 (2).

A direct comparison of our series with others is rather difficult given the small number of cases compared to other far more numerous series and substantially confirms already published data (8). Despite this, the distinctiveness of the context allows to highlight the most frequent hospital clinical pictures in the child with their temporal distribution.

A Korean single-center study described clinical features and persistence of viral RNA in 91 infants hospitalized after detection of SARS-CoV-2, regardless of their clinical pattern. No MIS-C cases appears in this series and symptomatic patients presented respiratory or influenza-like symptoms confirming our observation (37). The average persistence of viral RNA oscillated between 18 and 20 days regardless of the reported symptoms confirming as a positive swab sample in our MIS-C cases was compatible with an infection of three or more weeks earlier (37).

In conclusion, the analysis of children presenting at the first epicentre of European SARS-CoV-2 epidemic, at a time when health care measures aimed at mitigating its diffusion were not in place, allowed us to recognize a dual pattern of disease, related to a different pathophysiology of injury. The distinctive immune response that children mount against the virus seems to have paramount importance in this battle, and understanding its deepest features will probably contribute to an effective management of patients with COVID-19.

## Data Availability Statement

The original contributions presented in the study are included in the article/Supplementary Material further inquiries can be directed to the corresponding author/s.

## Ethics Statement

The studies involving human participants were reviewed and approved by Bergamo Ethics Committee. Written informed consent to participate in this study was provided by the participants' legal guardian/next of kin.

## Author's Note

Infection by SARS-CoV-2, named COVID-19, spread worldwide since the end of 2019 and Bergamo was one of the most affected areas in Europe. Following the first outbreak, more than half of the population of the Bergamo province had been infected, unfortunately becoming a model of the impact of COVID-19 epidemic on people's health. COVID-19 presents different patterns of clinical presentation in adults and children. In adults, the clinical picture is dominated by interstitial pneumonia possibly complicated by cardiovascular involvement. In children the clinical presentation is mild, mostly with an influenza-like pattern; serious cases are rare and full recovery is the rule. We performed an analysis of clinical patterns of paediatric patients admitted to our unit during the first three months of the outbreak. The peculiarity of our epidemiological situation can add new insights in characteristics of SARS-CoV-2 infection in children, following a period of unmitigated viral circulation. An immunological discussion is provided to fournish possible explanations about differences with adult patterns of the disease.

## Author Contributions

All authors listed have made a substantial, direct and intellectual contribution to the work, and approved it for publication.

## Conflict of Interest

The authors declare that the research was conducted in the absence of any commercial or financial relationships that could be construed as a potential conflict of interest.

## Abbreviations

SARS-CoV-2: severe acute respiratory syndrome coronavirus 2
COVID-19: coronavirus disease-19
MIS-C: Multisystem inflammatory syndrome in children
NP/OP: nasopharyngeal/oropharingeal
PPE: personal protective equipment
RT-PCR: reverse-transcriptase polymerase-chain-reaction
ICU: intensive care unit
IL-6: interleukin-6
PCR: polymerase chain reaction
WBC: white blood cells
IVIG: intravenous immunoglobulin
ASA: Acetyl salicylate acid
CRP: C-reactive protein
LDH: lactate dehydrogenase
ALT: Alanine aminotransferase
IQR: interquartile range
ACE-2: angiotensin converting enzyme-2
TGF-ß: transforming growth factor-ß
IL-10: Interleukin-10.
